# Adaptive Neural Network Control of Time Delay Teleoperation System Based on Model Approximation

**DOI:** 10.3390/s21227443

**Published:** 2021-11-09

**Authors:** Yaxiang Wang, Jiawei Tian, Yan Liu, Bo Yang, Shan Liu, Lirong Yin, Wenfeng Zheng

**Affiliations:** 1 School of Innovation and Entrepreneurship, Xi’an Fanyi University, Xi’an 710105, China; yaxiang.wang.cn@gmail.com; 2 School of Automation, University of Electronic Science and Technology of China, Chengdu 610054, China; jravis.tian23@gmail.com (J.T.); eevian.liu@gmail.com (Y.L.); yangbo.sd@gmail.com (B.Y.); wenfeng.zheng.cn@gmail.com (W.Z.); 3 Department of Geography and Anthropology, Louisiana State University, Baton Rouge, LA 70803, USA; yin.lyra@gmail.com

**Keywords:** neural network method, adaptive method, teleoperation system, time delay, force feedback, friction and disturbance

## Abstract

A bilateral neural network adaptive controller is designed for a class of teleoperation systems with constant time delay, external disturbance and internal friction. The stability of the teleoperation force feedback system with constant communication channel delay and nonlinear, complex, and uncertain constant time delay is guaranteed, and its tracking performance is improved. In the controller design process, the neural network method is used to approximate the system model, and the unknown internal friction and external disturbance of the system are estimated by the adaptive method, so as to avoid the influence of nonlinear uncertainties on the system.

## 1. Introduction

**Figure 1:**
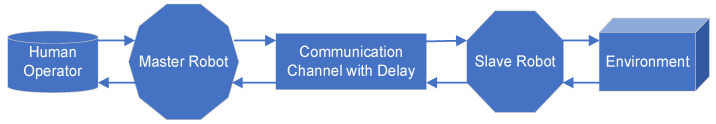
The control structure of the bilateral teleoperation robot system.

Teleoperation robot systems have developed rapidly and been applied to many fields, such as space robots [1], remote surgery robots [2,3], teleoperation mobile robots [4] and so on. The general remote operation robot system mainly includes: a master module, operator module, master controller, communication channel, slave controller, slave, environment and so on. The frame diagram is shown in Figure 1. However, in the actual teleoperation mechanical system, it is difficult to obtain accurate mechanical parameters of the robot, such as mass, length, center of mass or moment of inertia, etc., resulting in the system dynamics parameters (inertia vector matrix, centrifugal force matrix and gravity term matrix) not being accurate, as well as uncertain external interference and mechanical internal friction, which are common in robot workspace control [5]. The complex working environment or the robot’s mechanical structure is, therefore, more complicated or can be destroyed. After modeling using mathematical models, these may be random or time-varying nonlinear functions. Therefore, we cannot accurately establish the mathematical model of the system. That is, the mathematical model of the system contains uncertainty. These problems are often encountered in teleoperation systems, and their manifestations are quite variable. Moreover, the uncertainty of these teleoperation system models not only affects the performance of the system but also makes the entire system unstable [6]. Therefore, how to solve the above problems has been a wide concern in the field of control [7].

The methods proposed in reference [8,9] cannot effectively solve the problem of system model uncertainty in the form of time-varying functions. However, the neural network can approach the linear and nonlinear model by learning, so the neural network can be integrated into the system control. Therefore, the adaptive neural network and fuzzy control method [10,11,12,13] have good performance for the system with time delay, nonlinear control, complexity and uncertainty, and the method is widely used in the field of robot control. However, the number of adaptive laws depends on the number of neural network points. The accuracy of model approximation can be improved by increasing the number of neural network points. Thus, it takes a long time to learn online. Therefore, it is of great significance to design a bilateral neural network adaptive controller with short online learning time for the teleoperation system with time-delay force feedback [14,15,16].

Based on the above discussion and analysis, this paper designs a bilateral neural network adaptive controller for a class of teleoperation systems with constant time delay, external interference and mechanical internal friction, which is closer to the actual dynamic model of the teleoperation system. Finally, it guarantees the existence of constant communication channel delay, and nonlinear, complex and uncertain constant delay. The stability of the teleoperation force feedback system is improved and its tracking performance is improved. In the controller design process, the neural network method is used to approximate the system model, and the unknown internal friction and external disturbance of the system are estimated by the adaptive method so as to avoid the influence of nonlinear uncertainties on the system.

### 1.1. Spatial Dynamic Model of Master Slave–Robot Joint

Without considering the joint friction and external interference, the general dynamic equations of the master robot and the slave robot of the teleoperation system can be expressed by the following Euler–Lagrange Equation [17]

(1) $$M_{q_{m}}\left(q_{m}\right){\ddot{q}}_{m}+C_{q_{m}}\left(q_{m},{\dot{q}}_{m}\right){\dot{q}}_{m}+G_{q_{m}}(q_{m})=\tau_{m}+J_{m}^{T}\left(q_{m}\right)F_{h}$$

(2) $$M_{q_{s}}\left(q_{s}\right){\ddot{q}}_{s}+C_{q_{s}}\left(q_{s},{\dot{q}}_{s}\right){\dot{q}}_{s}+G_{q_{s}}(q_{s})=\tau_{s}-J_{s}^{T}\left(q_{s}\right)F_{e}$$

In order to simplify the description, we can see Table 1.

The dynamic equations of the master robot and slave robot in the teleoperation system [18], namely Equations (1) and (2), have the following properties:

**Property** **1.** *The inertia matrix* $M_{q_{i}}\left(q_{i}\right)$ *is symmetric and positive definite, with maximum and minimum values.*

(3) $$0<\lambda_{\min}\left\{M_{q_{i}}\left(q_{i}\right)\right\}I\leq M_{q_{i}}\left(q_{i}\right)\leq \lambda_{\max}\left\{M_{q_{i}}\left(q_{i}\right)\right\}I<\infty$$

**Property** **2.** *The matrix of Coriolis and centrifugal force* $C_{q_{i}}\left(q_{i},{\dot{q}}_{i}\right)$ *and satisfies:* ${\dot{M}}_{q_{i}}\left(q_{i}\right)-2C_{q_{i}}(q_{i},{\dot{q}}_{i})$ *is skew symmetric. Namely*

(4) $$\zeta^{T}\left({\dot{M}}_{q_{i}}\left(q_{i}\right)-2C_{q_{i}}(q_{i},{\dot{q}}_{i})\right)\zeta =0,\forall \zeta \in {ℜ}^{n\times 1}$$

**Property** **3.** *The terms on the left side of the dynamic Formulas (1) and (2) of the master robot and slave robot of the teleoperation system are transformed linearly, and the unknown constant parameter vector* $\theta_{d}={\left[\theta_{d1},\cdot \cdot \cdot ,\theta_{dr}\right]}^{T}$ *of the robot is defined as*

(5) $$M_{q_{i}}\left(q_{i}\right){\ddot{q}}_{i}+C_{q_{i}}\left(q_{i},{\dot{q}}_{i}\right){\dot{q}}_{i}+G_{q_{i}}(q_{i})=\tau_{i}=Y_{d}\left(q_{i},{\dot{q}}_{i},{\ddot{q}}_{i}\right)\theta_{d}$$

Among them, $Y_{d}\left(q_{i},{\dot{q}}_{i},{\ddot{q}}_{i}\right)\in {ℜ}^{n\times r}$ is called the dynamic regression matrix, which is the known function matrix about the joint variables of the robot.

### 1.2. Space Dynamic Model of Combined Teleoperation System Joint

We can observe that the dynamic model of the master and slave robot is in the joint space, and the dynamic model of the operator and the environment is in the working space. Thus, the dynamic model of the teleoperation system cannot be unified, so the dynamic model is increased. It is difficult to design a bilateral controller [19,20]. Therefore, it is necessary to use the master robot and slave robot workspace kinematics model to transform the dynamic model of the operator module and environment module workspace into joint space. Finally, the joint space dynamic model of the master robot and slave robot is sorted out, and then the simplified joint space model of the teleoperation system is obtained. The joint space dynamic model of the operator and environment is obtained as follows

(6) $$F_{h}=f_{h}^{*}-M_{h}J_{m}{\ddot{q}}_{m}-\left(B_{h}J_{m}+M_{h}{\dot{J}}_{m}\right){\dot{q}}_{m}-K_{h}h\left(q_{m}\right)$$

(7) $$\begin{array}{cc} F_{e}=f_{e}^{*}+M_{e}J_{s}{\ddot{q}}_{s}-\left(B_{e}J_{s}+M_{e}{\dot{J}}_{s}\right){\dot{q}}_{s}-K_{e}h\left(q_{s}\right) & \end{array}$$

The two sides of Equations (6) and (7) are multiplied by $J_{m}^{T}$ and substituting into Equations (1) and (2), respectively, and the simplified joint space model of the teleoperation system is obtained

(8) $$M_{i}\left(q_{i}\right){\ddot{q}}_{i}+C_{i}\left(q_{i},{\dot{q}}_{i}\right){\dot{q}}_{i}+G_{i}\left(q_{i}\right)=\tau_{i},\;i=m,s$$

among which

(9) $$M_{m}\left(q_{m}\right)=M_{q_{m}}\left(q_{m}\right)+J_{m}^{T}M_{h}J_{m}$$

(10) $$C_{m}\left(q_{m},{\dot{q}}_{m}\right)=C_{q_{m}}\left(q_{m},{\dot{q}}_{m}\right)+J_{m}^{T}B_{h}J_{m}+J_{m}^{T}M_{h}{\dot{J}}_{m}$$

(11) $$G_{m}\left(q_{m}\right)=G_{q_{m}}\left(q_{m}\right)+J_{m}^{T}K_{h}h_{m}\left(q_{m}\right)-J_{m}^{T}f_{h}^{*}$$

(12) $$M_{s}\left(q_{s}\right)=M_{q_{s}}\left(q_{s}\right)+J_{s}^{T}M_{e}J_{s}$$

(13) $$C_{s}\left(q_{s},{\dot{q}}_{s}\right)=C_{q_{s}}\left(q_{s},{\dot{q}}_{s}\right)+J_{s}^{T}B_{e}J_{s}+J_{s}^{T}M_{e}{\dot{J}}_{s}$$

(14) $$G_{s}\left(q_{s}\right)=G_{q_{s}}\left(q_{s}\right)+J_{s}^{T}K_{e}h_{s}\left(q_{s}\right)+J_{s}^{T}f_{e}^{*}$$

After unifying the dynamics of each module in the teleoperation system into the joint space, according to Property 1–3, we can deduce the mathematical models of the combined teleoperation system, Equation (8) has the following new properties: for all, they represent the master and the slave.

**Property** **4.** *The inertial matrix* $M_{i}\left(q_{i}\right)$ *is symmetric and positive definite, with maximum and minimum values.*

(15) $$0<\lambda_{\min}\left\{M_{i}\left(q_{i}\right)\right\}I\leq M_{i}\left(q_{i}\right)\leq \lambda_{\max}\left\{M_{i}\left(q_{i}\right)\right\}I<\infty$$

**Property** **5.** *For* $\forall \xi \in {ℜ}^{n\times 1}$ *, the Coriolis matrix* ${\dot{M}}_{i}\left(q_{i}\right)$ *and the centrifugal force matrix* $C_{i}\left(q_{i},{\dot{q}}_{i}\right)$ *satisfy*

(16) $$\xi^{T}\left({\dot{M}}_{i}\left(q_{i}\right)-2C_{i}(q_{i},{\dot{q}}_{i})\right)\xi =-2\xi^{T}B_{i}\zeta$$

**Property** **6.** *Unify the mathematical models of each module in the teleoperation system into the joint space and substitute them into the joint space mathematical models of the master robot and the slave robot. After sorting out the dynamic models, the items on the left of Equation (8) are obtained. The unknown constant parameter vector of the robot can also be defined as* $\theta_{z}={\left[\theta_{z1},\cdot \cdot \cdot ,\theta_{zr}\right]}^{T}$ *. After linear transformation, it is obtained that the parameter vector* $\theta_{z}$ *of the robot is linear*

(17) $$M_{i}\left(q_{i}\right){\ddot{q}}_{i}+C_{i}\left(q_{i},{\dot{q}}_{i}\right){\dot{q}}_{i}+G_{i}(q_{i})=\tau_{i}=Y_{z}\left(q_{i},{\dot{q}}_{i},{\ddot{q}}_{i}\right)\theta_{z}$$

where, i=m,s , $Y_{z}\left(q_{i},{\dot{q}}_{i},{\ddot{q}}_{i}\right)\in {ℜ}^{n\times r}$ is called the dynamic regression matrix, which is the known function matrix about the joint variables of the robot.

## 2. Materials and Methods

### 2.1. Problem Statement

The main goal of this paper is to consider the existence of mechanical internal friction and external friction between master robot and slave robot in teleoperation system. Based on the position error control structure, a bilateral controller is designed for the teleoperation system to make the slave robot follow the position signal of the master robot, to improve the performance and ensure the stability of the system. The RBF (Radial Basis Function) neural network (RBFNN) is used to approximate the system model because RBFNN is a single hidden layer, feedforward neural network based on function approximation proposed in the late 1980s.

At present, the commonly used function approximation methods are neural network and fuzzy system approximation methods [21], namely neural network adaptive control and fuzzy adaptive control. In the process of control law design, Lyapunov’s direct method is used to design control law and adaptive law. By designing appropriate adaptive law parameters, the stability and convergence of the whole closed-loop nonlinear system are guaranteed. At present, there are two kinds of radial basis function and multi-layer neural networks. Through comparative analysis, it can be found that the former can approximate any nonlinear function, and can solve the problem that the system cannot be established by a mathematical model under the condition of fewer network points, and has good generalization ability and fast learning convergence speed. To sum up, this paper chooses RBF neural network, and the system dynamic model contains time delay signal [22].

The unknown friction and external disturbance of each joint between the master robot and the slave robot are taken into account in the teleoperation system studied in this chapter. Therefore, according to the general joint space dynamic model of the combined teleoperation system described in Section 1.2, Equations (35) and (36) can be used to describe a joint space dynamic model with internal friction The mathematical model of teleoperation system with external interference is as follows

(18) $$M_{i}\left(q_{i}\right){\ddot{q}}_{i}+C_{i}\left(q_{i},{\dot{q}}_{i}\right){\dot{q}}_{i}+G_{i}\left(q_{i}\right)+f_{i}\left(q_{i},{\dot{q}}_{i}\right)+f_{ci}\left({\dot{q}}_{i}\right)=\tau_{i},\;i=m,s$$

Among them, $f_{cm}\left({\dot{q}}_{m}\right),f_{cs}\left({\dot{q}}_{s}\right)\in {ℜ}^{n\times 1}$ represents the mechanical internal friction of the master robot and the slave robot is bounded, and $f_{m}\left(q_{m},{\dot{q}}_{m}\right),f_{s}\left(q_{s},{\dot{q}}_{s}\right)\in {ℜ}^{n\times 1}$ represents the uncertain bounded external interference of the master robot and the slave robot, respectively [23].

In the teleoperation system based on position error control structure, there is a constant time delay $T_{m},T_{s}$ in the forward communication channel and the reverse communication channel.

(19) $$e_{m}=q_{s}\left(t-T_{s}\right)-q_{m}\left(t\right)$$

(20) $$e_{s}=q_{m}\left(t-T_{m}\right)-q_{s}\left(t\right)$$

The goal of the time-delay force feedback teleoperation system is to keep the control torque of the master robot and the slave robot bounded so as to ensure that the position tracking error of the master–slave robot in the system can converge to zero and make the system stable. Therefore, the sliding mode function is defined as

(21) $$r_{m}={\dot{e}}_{m}+\Lambda_{m}e_{m}$$

(22) $$r_{s}={\dot{e}}_{s}+\Lambda_{s}e_{s}$$

where $\Lambda_{j}=\Lambda_{j}^{T}>0,j=m,s$ , which is a constant diagonal matrix.

By substituting Equations (21) and (22) into Equation (18), the following results are obtained

(23) $$M_{i}\left(q_{i}\right){\ddot{r}}_{i}+C_{i}\left(q_{i},{\dot{q}}_{i}\right){\dot{r}}_{i}={\tilde{f}}_{i}\left(X_{i}\right)-\tau_{i}+f_{i}\left(q_{i},{\dot{q}}_{i}\right)+f_{ci}\left({\dot{q}}_{i}\right),\;i=m,s$$

Here, ${\tilde{f}}_{m}\left(X_{m}\right)$ and ${\tilde{f}}_{s}\left(X_{s}\right)$ are the uncertainties of the master robot model and the slave robot model of the system

(24) $$\begin{array}{l} {\tilde{f}}_{m}\left(X_{m}\right)=M_{m}\left(q_{m}\right){\ddot{q}}_{s}\left(t-T_{s}\right)+M_{m}\left(q_{m}\right)\Lambda_{m}{\dot{e}}_{m}+C_{m}\left(q_{m},{\dot{q}}_{m}\right){\dot{q}}_{s}\left(t-T_{s}\right)\\ \begin{array}{cccc} & & & + \end{array}C_{m}\left(q_{m},{\dot{q}}_{m}\right)\Lambda_{m}e_{m}+G_{m}\left(q_{m}\right) \end{array}$$

(25) $$\begin{array}{l} {\tilde{f}}_{s}\left(X_{s}\right)=M_{s}\left(q_{s}\right){\ddot{q}}_{m}\left(t-T_{m}\right)+M_{s}\left(q_{s}\right)\Lambda_{s}{\dot{e}}_{s}+C_{s}\left(q_{s},{\dot{q}}_{s}\right){\dot{q}}_{m}\left(t-T_{m}\right)\\ \begin{array}{cccc} & & & + \end{array}C_{s}\left(q_{s},{\dot{q}}_{s}\right)\Lambda_{s}e_{s}+G_{s}\left(q_{s}\right) \end{array}$$

Usually, the uncertainty ${\tilde{f}}_{j}\left(X_{j}\right),j=m,s$ of teleoperation system model is nonlinear and unknown. Therefore, in order to make the teleoperation system have practical significance, we can use the method of model approximation to obtain the approximate value of the uncertainty ${\tilde{f}}_{j}\left(X_{j}\right)$ so as to solve the above problems. In the control of nonlinear systems, the performance of the controller can be improved by using function approximation method when there are nonlinear uncertainties [23]. This chapter chooses RBF neural network to approximate the system model uncertainty ${\tilde{f}}_{j}\left(X_{j}\right)$ .

### 2.2. Preliminary Knowledge

The following is the design and stability analysis of the bilateral controller of the teleoperation system with time-delay force feedback.

We choose to use RBFNN method to approximate the system model. Therefore, according to the Equations (24) and (25) of the uncertainties ${\tilde{f}}_{j}\left(X_{j}\right)$ of the system model, we can select the network input signals of the master controller and the slave controller as follows

(26) $$X_{m}={\left[{\ddot{q}}_{s}\left(t-T_{s}\right),{\dot{q}}_{s}\left(t-T_{s}\right),q_{s}\left(t-T_{s}\right),{\dot{q}}_{m}\left(t\right),q_{m}\left(t\right)\right]}^{T}$$

(27) $$X_{s}={\left[{\ddot{q}}_{m}\left(t-T_{m}\right),{\dot{q}}_{m}\left(t-T_{m}\right),q_{m}\left(t-T_{m}\right),{\dot{q}}_{s}\left(t\right),q_{s}\left(t\right)\right]}^{T}$$

RBFNN consists of two layers: the hidden layer, which is used to project the input network signal into a high-dimensional space, and the output layer, which is used to output the linear combination of output signals of hidden layer with adaptive parameter weight adjustment. It is a linear parameterized neural network and has excellent approximation performance. Suppose the uncertain continuous function is $F_{0}\left(X\right):{ℜ}^{p}\to ℜ$ approximated by RBFNN

(28) $$F_{nn}\left(X\right)=W_{0}^{T}\sigma_{0}\left(X\right)$$

**Figure 2:**
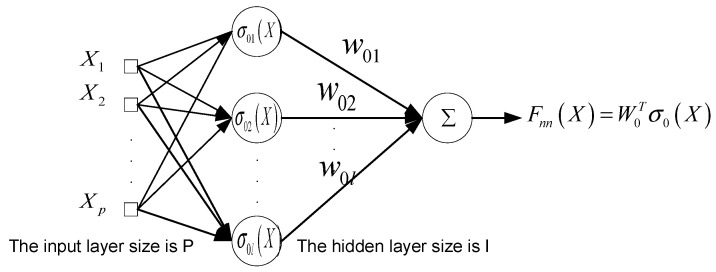
Schematic diagram of RBFNN working principle.

$X\in \Omega_{X}\in {ℜ}^{p}$ represents the network input vector, $W_{0}^{T}=\left[w_{01},w_{02},\cdot \cdot \cdot ,w_{0l}\right]\in {ℜ}^{l}$ represents the weight of the parameters to be adjusted, here l>1 represents the number of neural network points, and $\sigma_{0}\left(X\right)={\left[\sigma_{01}\left(X\right),\sigma_{02}\left(X\right),\cdot \cdot \cdot ,\sigma_{0l}\left(X\right)\right]}^{T}$ represents the Gaussian basis functions, which are defined as

(29) $$\sigma_{0}\left(X\right)=e^{-\frac{{\left(X-v_{i}\right)}^{T}\left(X-v_{i}\right)}{\eta^{2}}},i=1,\cdot \cdot \cdot ,l$$

where, $v_{i}\in \Omega_{X}$ and η>0 denote the center and width of the Gaussian function, respectively. The working principle of RBFNN is shown in Figure 2.

The conclusion of Reference [7] shows that for a compact set $\Omega_{X}\in {ℜ}^{p}$ , when it is large enough, the radial basis function neural network of Equation (19) can arbitrarily approximate the uncertain continuous function $F_{0}\left(X\right)$ . For any accuracy rate ε>0 , there are

(30) $$F_{0}\left(X\right)=W_{0}^{*T}\sigma_{0}\left(X\right)+\delta_{0}\left(X\right)$$

where $X\in \Omega_{X}\in {ℜ}^{p}$ is the estimation error, its upper boundary is ε , and $W_{0}^{*}$ is the optimal weight vector and satisfies the following conditions

(31) $$W_{0}^{*}:=\begin{array}{cc} \arg & \underset{W_{0}\in {ℜ}^{l}}{\min} \end{array}\left\{\underset{X\in \Omega_{X}}{\sup}\left|F_{0}\left(X\right)-W_{0}^{T}\sigma_{0}\left(X\right)\right|\right\}$$

For the radial basis function neural network Equation (30), which contains Gaussian function Equation (31), assuming $\rho :=\frac{1}{2}\min_{i\neq j}\left\|v_{i}-v_{j}\right\|$ , the following inequality is established.

(32) $$\left\|\sigma_{0}\left(X\right)\right\|\leq {\sum_{m=0}^{\infty }3p\left(m+2\right)}^{p-1}e^{-2\rho^{2}m^{2}/\eta^{2}}:=s$$

where s is the finite value, m is the number of convergent infinite sequence terms $\left\{3p{\left(m+2\right)}^{p-1}e^{-2\rho^{2}m^{2}/\eta^{2}}\right\}$ .

### 2.3. Design and Stability Analysis of Bilateral Controller

Firstly, for the telerobot system introduced in Section 2.2, we defined a high calculus Lyapunov–Krasovskii candidate function V(t) that satisfies the following requirements, and which is used to analyze the stability and location tracking performance of the operating system

(33) $$V\left(t\right)=\frac{1}{2}r_{m}^{T}M_{m}\left(q_{m}\right)r_{m}+\frac{1}{2}r_{s}^{T}M_{s}\left(q_{s}\right)r_{s}+\frac{1}{2\lambda_{m}}{\tilde{\theta }}_{m}^{2}+\frac{1}{2\lambda_{s}}{\tilde{\theta }}_{s}^{2}+\frac{1}{2\gamma_{m}}{\tilde{d}}_{m}^{2}+\frac{1}{2\gamma_{s}}{\tilde{d}}_{s}^{2}$$

where let, j=m,s , ${\tilde{\theta }}_{j}=\theta_{j}-{\hat{\theta }}_{j}$ , ${\tilde{d}}_{j}=d_{j}-{\hat{d}}_{j}$ , $\lambda_{j},\gamma_{j}>0$ be a constant.

By deriving the two sides of Equation (33), $\dot{V}\left(t\right)$ is obtained by

(34) $$\dot{V}\left(t\right)=\frac{1}{2}r_{m}^{T}{\dot{M}}_{m}\left(q_{m}\right)r_{m}+r_{m}^{T}M_{m}\left(q_{m}\right){\dot{r}}_{m}+\frac{1}{2}r_{s}^{T}{\dot{M}}_{s}\left(q_{s}\right)r_{s}+r_{s}^{T}M_{s}\left(q_{s}\right){\dot{r}}_{s}\;+\frac{1}{\lambda_{m}}{\tilde{\theta }}_{m}{\dot{\tilde{\theta }}}_{m}+\frac{1}{\lambda_{s}}{\tilde{\theta }}_{s}{\dot{\tilde{\theta }}}_{s}+\frac{1}{\gamma_{m}}{\tilde{d}}_{m}{\dot{\tilde{d}}}_{m}+\frac{1}{\gamma_{s}}{\tilde{d}}_{s}{\dot{\tilde{d}}}_{s}$$

From Property 5, $r_{j}^{T}\left({\dot{M}}_{j}\left(q_{j}\right)-2C_{j}(q_{j},{\dot{q}}_{j})\right)r_{j}=-2r_{j}^{T}B_{i}r_{j}^{T},j=m,s,i=h,e$ . Again, by estimation error of $\theta_{j}$ , ${\tilde{\theta }}_{j}=\theta_{j}-{\hat{\theta }}_{j}$ where ${\dot{\tilde{\theta }}}_{j}=-{\dot{\hat{\theta }}}_{j}$ by estimation error of $d_{j}$ , ${\tilde{d}}_{j}=d_{j}-{\hat{d}}_{j}$ where ${\dot{\tilde{d}}}_{j}=-{\dot{\hat{d}}}_{j}$ . By substituting the above Equation into Equation (34), we can obtain the following results

(35) $$\begin{array}{l} \dot{V}\left(t\right)=r_{m}^{T}\left({\tilde{f}}_{m}\left(X_{m}\right)-\tau_{m}+f_{cm}({\dot{q}}_{m})+f_{m}\left(q_{m},{\dot{q}}_{m}\right)\right)-2r_{m}^{T}B_{h}\left(q_{m}\right)r_{m}\\ \begin{array}{ccc} & & +r_{s}^{T}\left({\tilde{f}}_{s}\left(X_{s}\right)-\tau_{s}+f_{cs}({\dot{q}}_{s})+f_{s}\left(q_{s},{\dot{q}}_{s}\right)\right) \end{array}-2r_{s}^{T}B_{e}\left(q_{m}\right)r_{s} \end{array}\;-\frac{1}{\lambda_{m}}{\tilde{\theta }}_{m}{\dot{\hat{\theta }}}_{m}-\frac{1}{\lambda_{s}}{\tilde{\theta }}_{m}{\dot{\hat{\theta }}}_{m}-\frac{1}{\gamma_{m}}{\tilde{d}}_{m}{\dot{\hat{d}}}_{m}-\frac{1}{\gamma_{s}}{\tilde{d}}_{s}{\dot{\hat{d}}}_{s}$$

By using radial basis function neural network $W_{j}^{T}\sigma_{j}\left(X_{j}\right)\in {ℜ}^{n}$ , $W_{j}\in {ℜ}^{n\times n}$ , the uncertain term ${\tilde{f}}_{j}\left(X_{j}\right),j=m,s$ is approximated

(36) $${\tilde{f}}_{j}\left(X_{j}\right)=W_{j}^{T}\sigma_{j}\left(X_{j}\right)+\delta_{j}\left(X_{j}\right)$$

where $\delta_{j}\left(X_{j}\right)$ is the estimation error that satisfies $\left\|\delta_{j}\left(X_{j}\right)\right\|\leq \varepsilon_{j}$ where $\varepsilon_{j}>0$ , which is the constant.

By substituting Equation (36) into Equation (35), the following results are obtained

(37) $$\begin{array}{l} \dot{V}\left(t\right)=r_{m}^{T}\left(W_{m}^{T}\sigma_{m}\left(X_{m}\right)+\delta_{m}\left(X_{m}\right)-\tau_{m}+f_{cm}({\dot{q}}_{m})+f_{m}\left(q_{m},{\dot{q}}_{m}\right)\right)\\ \begin{array}{ccc} & & +r_{s}^{T}\left(W_{s}^{T}\sigma_{s}\left(X_{s}\right)+\delta_{s}\left(X_{s}\right)-\tau_{s}+f_{cs}({\dot{q}}_{s})+f_{s}\left(q_{s},{\dot{q}}_{s}\right)\right) \end{array} \end{array}\;-2r_{m}^{T}B_{h}r_{m}-2r_{s}^{T}B_{e}r_{s}-\frac{1}{\lambda_{m}}{\tilde{\theta }}_{m}{\dot{\hat{\theta }}}_{m}-\frac{1}{\lambda_{s}}{\tilde{\theta }}_{s}{\dot{\hat{\theta }}}_{s}-\frac{1}{\gamma_{m}}{\tilde{d}}_{m}{\dot{\hat{d}}}_{m}-\frac{1}{\gamma_{s}}{\tilde{d}}_{s}{\dot{\hat{d}}}_{s}$$

By using the properties of matrix norm inequality, the following inequalities can be obtained

(38) $$r_{j}^{T}W_{j}^{T}\sigma_{j}\left(X_{j}\right)\leq \left\|r_{j}\right\|{\left\|W_{j}\right\|}_{F}\left\|\sigma_{j}\left(X_{j}\right)\right\|\leq \frac{1}{2a_{j}^{2}}r_{j}^{T}r_{j}\theta_{j}\sigma_{j}^{T}\sigma_{j}+\frac{1}{2}a_{j}^{2}$$

where, let $\theta_{j}={\left\|W_{j}\right\|}_{F}^{2}$ be the weight of the radial basis function to be estimated and adjusted, $a_{j}$ is a normal number. According to Properties 2–4, the internal friction $f_{cj}({\dot{q}}_{j})$ between master robot and slave robot in the system is continuous and bounded, and the unknown external interference $f_{j}(q_{j},{\dot{q}}_{j})$ of master robot and slave robot is continuous bounded, assuming $d_{j}\geq \left(\left\|\delta_{j}\left(X_{j}\right)\right\|+\left\|f_{cj}({\dot{q}}_{j})\right\|+\left\|f_{j}(q_{j},{\dot{q}}_{j})\right\|\right)$ j=m,s , then

(39) $$\begin{array}{l} r_{j}^{T}\left(\delta_{j}\left(X_{j}\right)+f_{cj}({\dot{q}}_{j})+f_{j}(q_{j},{\dot{q}}_{j})\right)\\ \begin{array}{cc} & \leq \left\|r_{j}\right\| \end{array}\left(\left\|\delta_{j}\left(X_{j}\right)\right\|+\left\|f_{cj}({\dot{q}}_{j})\right\|+\left\|f_{j}(q_{j},{\dot{q}}_{j})\right\|\right)\leq \left\|r_{j}\right\|d_{j} \end{array}$$

Substituting Equations (38) and (29) into Lyapunov derivative Equation (37), we obtain the following results

(40) $$\dot{V}\left(t\right)\leq \sum_{i=m,s}\left(r_{i}^{T}\left(\frac{1}{2a_{i}^{2}}r_{i}\theta_{i}\sigma_{i}^{T}\sigma_{i}-\tau_{i}\right)+\left\|r_{j}\right\|d_{j}+\frac{1}{2}a_{j}^{2}\right)-2r_{m}^{T}B_{h}r_{m}\;-2r_{s}^{T}B_{e}r_{s}-\frac{1}{\lambda_{m}}{\tilde{\theta }}_{m}{\dot{\hat{\theta }}}_{m}-\frac{1}{\lambda_{s}}{\tilde{\theta }}_{s}{\dot{\hat{\theta }}}_{s}-\frac{1}{\gamma_{m}}{\tilde{d}}_{m}{\dot{\hat{d}}}_{m}-\frac{1}{\gamma_{s}}{\tilde{d}}_{s}{\dot{\hat{d}}}_{s}$$

We can design the neural network adaptive control law of time delay teleoperation system based on model approximation

(41) $$\tau_{j}=k_{j}r_{j}+\frac{r_{j}}{2a_{j}^{2}}{\hat{\theta }}_{j}\sigma_{j}^{T}\sigma_{j}+\frac{r_{j}}{\left\|r_{j}\right\|+e^{-a_{j}t}}{\hat{d}}_{j}$$

Here, j=m,s , $k_{j}$ is the normal number. Thus, from Equation (41), there are

(42) $$-r_{j}^{T}\tau_{j}=-k_{j}r_{j}^{T}c_{j}r_{j}-\frac{1}{2a_{j}^{2}}r_{j}^{T}c_{j}r_{j}{\hat{\theta }}_{j}\sigma_{j}^{T}\sigma_{j}-\frac{1}{\left\|r_{j}\right\|+e^{-a_{j}t}}r_{j}^{T}c_{j}r_{j}{\hat{d}}_{j}$$

By substituting Equation (42) into Equation (40), the following results are obtained

(43) $$\begin{array}{l} \dot{V}\left(t\right)\leq -k_{m}r_{m}^{T}r_{m}-k_{s}r_{s}^{T}r_{s}+\frac{1}{2}a_{m}^{2}+\frac{1}{2}a_{s}^{2}-2r_{m}^{T}B_{h}r_{m}-2r_{s}^{T}B_{e}r_{s}\\ \begin{array}{ccc} & & +\sum_{i=m,s}\left(\frac{1}{\lambda_{i}}{\tilde{\theta }}_{i}\left(\frac{\lambda_{i}}{2a_{i}^{2}}r_{i}^{T}r_{i}\sigma_{i}^{T}\sigma_{i}-{\dot{\hat{\theta }}}_{i}\right)+\frac{1}{\gamma_{i}}{\tilde{d}}_{i}\left({\frac{\gamma_{i}r_{i}^{T}r_{i}}{\left\|r_{i}\right\|+e^{-a_{i}t}}}_{s}-{\dot{\hat{d}}}_{i}\right)\right) \end{array} \end{array}\;+\left\|r_{m}\right\|d_{m}+\left\|r_{s}\right\|d_{s}-\frac{r_{m}^{T}r_{m}}{\left\|r_{m}\right\|+e^{-a_{m}t}}d_{m}-\frac{r_{s}^{T}r_{s}}{\left\|r_{s}\right\|+e^{-a_{s}t}}d_{s}$$

Therefore, we can design the neural network adaptive controller of the master robot and the slave robot based on model approximation

(44) $${\dot{\hat{\theta }}}_{j}=\frac{\lambda_{j}}{2a_{j}^{2}}r_{j}^{T}r_{j}\sigma_{j}^{T}\sigma_{j}-\psi_{j}{\hat{\theta }}_{j}$$

(45) $${\dot{\hat{d}}}_{j}=\frac{\gamma_{j}r_{j}^{T}r_{j}}{\left\|r_{j}\right\|+e^{-a_{j}t}}-\upsilon_{j}{\hat{d}}_{j}$$

where $\psi_{j}$ , $\upsilon_{j}$ are the normal number. By substituting the Adaptive Law (44) and Equation (45) into Equation (43), the following results are obtained

(46) $$\dot{V}\left(t\right)\leq -k_{m}r_{m}^{T}r_{m}-k_{s}r_{s}^{T}r_{s}+\frac{1}{2}a_{m}^{2}+\frac{1}{2}a_{s}^{2}-2r_{m}^{T}B_{h}r_{m}-2r_{s}^{T}B_{e}r_{s}\;+\sum_{i=m,s}\left(\frac{\psi_{i}}{\lambda_{i}}{\tilde{\theta }}_{i}{\hat{\theta }}_{i}+\frac{\upsilon_{i}}{\gamma_{i}}{\tilde{d}}_{i}{\hat{d}}_{i}+\left\|r_{i}\right\|d_{i}-\frac{r_{i}^{T}r_{i}}{\left\|r_{i}\right\|+e^{-a_{i}t}}d_{i}\right)$$

**Figure 3:**
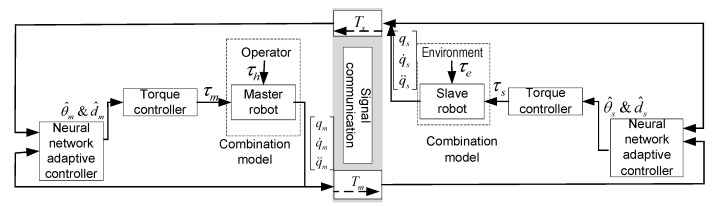
Adaptive neural network control block diagram of time delay teleoperation system based on model approximation.

Therefore, the time-delay force feedback teleoperation system based on model approximation in this paper includes a bilateral position control closed-loop, and the adaptive neural network control block diagram is shown in Figure 3. As can be seen from the figure, we combine the operator model with the master robot, and the environment model with the slave robot. In order to simplify the processed teleoperation system, two-sided controller Equation (41) and adaptive estimation law Equations (44) and (45) are designed to ensure the system stability. At the same time, this can solve the problems of time delay and nonlinear uncertainties of the system model, so as to improve the tracking performance and instantaneous performance of Equation (18) of time-delay force feedback teleoperation systems based on position error.

The stability of the closed-loop teleoperation system with time-delay force feedback, as shown in Figure 3, is discussed, and the position tracking performance between the master robot and the slave robot is analyzed.

In the case of contact, the closed-loop time-delay force feedback teleoperation system is controlled by two-sided neural network adaptive controller Equation (41) and adaptive law Equations (44) and (45). For this teleoperation system, the communication channel has constant time delay $T_{m},T_{s}$ .

It is stable under the neural network adaptive bilateral control law and adaptive law; that is, the tracking errors $e_{m},e_{s}$ are bounded at the joint space robot’s speed ${\dot{q}}_{m},{\dot{q}}_{s}$ , and when the time approaches infinity, the velocity ${\dot{q}}_{m},{\dot{q}}_{s}$ and tracking errors $e_{m},e_{s}$ of the joint space robot converge to a small area where it is close to zero.

For proof, from ${\tilde{\theta }}_{j}=\theta_{j}-{\hat{\theta }}_{j}$ and ${\tilde{d}}_{j}=d_{j}-{\hat{d}}_{j}$ , j=m,s , there are

(47) $${\tilde{\theta }}_{j}{\hat{\theta }}_{j}={\tilde{\theta }}_{j}\left(\theta_{j}-{\tilde{\theta }}_{j}\right)\leq \frac{1}{2}\theta_{j}^{2}-\frac{1}{2}{\tilde{\theta }}_{j}^{2}$$

(48) $${\tilde{d}}_{j}{\hat{d}}_{j}={\tilde{d}}_{j}\left(d_{j}-{\tilde{d}}_{j}\right)\leq \frac{1}{2}d_{j}^{2}-\frac{1}{2}{\tilde{d}}_{j}^{2}$$

By substituting Equations (47) and (48) into Equation (46), the results are as follows

(49) $$\dot{V}\left(t\right)\leq -\sum_{i=h,e,j=m,s}\left(k_{j}r_{j}^{T}r_{j}+2r_{j}^{T}B_{i}r_{j}+\frac{\psi_{j}}{2\lambda_{j}}{\tilde{\theta }}_{j}^{2}+\frac{\upsilon_{j}}{2\gamma_{j}}{\tilde{d}}_{j}^{2}\right)\;\begin{array}{ccc} & & +\sum_{j=m,s}\left(\frac{\psi_{j}}{2\lambda_{j}}\theta_{j}^{2}+\frac{\upsilon_{j}}{2\gamma_{j}}d_{j}^{2}+e^{-a_{j}t}d_{j}+\frac{1}{2}a_{j}^{2}\right) \end{array}$$

where $a_{0}=\min\left\{\frac{2k_{j}}{\lambda_{m}\left(M_{j}\left(q_{j}\right)\right)},\psi_{j},\upsilon_{j},j=m,s\right\}$ , and $b_{0}=\sum_{j=m,s}\left(\frac{\psi_{j}}{2\lambda_{j}}\theta_{j}^{2}+\frac{\upsilon_{j}}{2\gamma_{j}}d_{j}^{2}+\frac{1}{2}a_{j}^{2}+d_{j}\right)$ and if $B_{h},B_{e}$ is a positive definite constant diagonal matrix, then Equation (49) can be written as follows

(50) $$\dot{V}\left(t\right)\leq a_{0}V+b_{0}$$

The Equation (50) is integrated as follows

(51) $$V\left(t\right)\leq \left(V\left(0\right)-\frac{b_{0}}{a_{0}}\right)e^{-a_{0}t}+\frac{b_{0}}{a_{0}}$$

When t→∞ , there were $V\left(t\right)\leq \frac{b_{0}}{a_{0}}$ . Finally, we achieve the following results

(52) $$\left\|r_{j}\right\|\leq \sqrt{\frac{2b_{0}}{\lambda_{m}\left(M_{j}\left(q_{j}\right)\right)a_{0}}},t\to \infty ,j=m,s$$

To sum up, by selecting appropriate parameters, when the time approaches infinity, the sliding mode function $r_{m},r_{s}$ can approach to the sliding mode of small area of 0, and the teleoperation system is stable under the bilateral neural network adaptive control law and adaptive law. That is, the tracking error $e_{m},e_{s}$ is bounded at the joint space robot’s speed ${\dot{q}}_{m},{\dot{q}}_{s}$ , and when the time approaches infinity, the velocity ${\dot{q}}_{m},{\dot{q}}_{s}$ and tracking error $e_{m},e_{s}$ of the joint space robot converge to a small area close to zero.

## 3. Results

In order to verify the effectiveness of the above control algorithm, the master robot and slave robot are simulated in the presence of contact with the operator and the environment. Simulink is used for simulation verification, and the S-function is used to establish the system model, and then the neural network adaptive control closed-loop system of the time-delay force feedback teleoperation system based on model approximation is built, as shown in Figure 3.

Considering the external interference and the internal friction of the robot, the dynamic models of the teleoperation system are Equation (18). In this paper, the master robot and slave robot in the teleoperation system adopt the 2-DOF, 2-link, rotary joint manipulator robot. Here, for the sake of simplicity and generality, the moment of inertia of the rod is ignored. The mathematical model of joint space dynamics is as follows

(53) $$M_{q_{i}}\left(q_{i}\right)=\left[\begin{array}{cc} m_{i1}l_{i1}^{2}+m_{i2}l_{i1}^{2}+m_{i2}l_{i2}^{2}+2m_{i2}l_{i1}l_{i2}\cos\left(q_{i2}\right) & m_{i2}l_{i2}^{2}+m_{i2}l_{i1}l_{i2}\cos\left(q_{i2}\right)\\ m_{i2}l_{i2}^{2}+m_{i2}l_{i1}l_{i2}\cos\left(q_{i2}\right) & m_{i2}l_{i2}^{2} \end{array}\right]$$

(54) $$C_{q_{i}}\left(q_{i},{\dot{q}}_{i}\right)=\left[\begin{array}{cc} -m_{i2}l_{i1}l_{i2}{\dot{q}}_{i2}\cos\left(q_{i2}\right) & -m_{i2}l_{i1}l_{i2}\left({\dot{q}}_{i1}+{\dot{q}}_{i2}\right)\sin\left(q_{i2}\right)\\ m_{i2}l_{i1}l_{i2}{\dot{q}}_{i1}\sin\left(q_{i2}\right) & 0 \end{array}\right]$$

(55) $$G_{q_{i}}(q_{i})=\left[\begin{array}{c} \left(m_{i1}l_{i2}+m_{i2}l_{i1}\right)g\cos\left(q_{i1}\right)+m_{i2}l_{i2}g\cos\left(q_{i1}+q_{i2}\right)\\ m_{i2}l_{i2}g\cos\left(q_{i1}+q_{i2}\right) \end{array}\right]$$

In addition, in the experiment, the external interference of the master robot and the slave robot is set as $f_{i}\left(q_{i},{\dot{q}}_{i}\right)={\left[\begin{array}{cc} 0.1q_{i1}{\dot{q}}_{i1}\sin\left(t\right) & 0.1q_{i2}{\dot{q}}_{i2}\sin\left(t\right) \end{array}\right]}^{T}$ . Set the internal friction of the master robot and the slave robot as $f_{cm}({\dot{q}}_{m})={\left[\begin{array}{cc} f_{d1}{\dot{q}}_{m1}+k_{1}sign\left({\dot{q}}_{m1}\right) & f_{d2}{\dot{q}}_{m2}+k_{2}sign\left({\dot{q}}_{m2}\right) \end{array}\right]}^{T}$ Here, $f_{d1},f_{d2},k_{1},k_{2}$ is a constant, and $f_{cs}({\dot{q}}_{s})={\left[\begin{array}{cc} f_{d3}{\dot{q}}_{s1}+k_{3}sign\left({\dot{q}}_{s1}\right) & f_{d4}{\dot{q}}_{s2}+k_{4}sign\left({\dot{q}}_{s2}\right) \end{array}\right]}^{T}$ where $f_{d1},f_{d2},k_{1},k_{2}$ are constant.

At the same time, the external force from the operator is selected as $f_{h}^{*}={\left[\begin{array}{cc} 25\left(1-\cos\left(0.05\pi t\right)\right) & 0 \end{array}\right]}^{T}$ , and the external force from the interaction between robot and environment is selected as $f_{e}^{*}={\left[\begin{array}{cc} 0 & 0 \end{array}\right]}^{T}$ .

In the process of building a closed-loop teleoperation system, the mechanical constant parameters related to the dynamics of the master robot, slave robot, operator and environment are shown in Table 2.

In the simulation, the initial position of the master robot and slave robot is set as $q_{m}\left(0\right)={\left[\begin{array}{cc} 0.4pi & 0.2pi \end{array}\right]}^{T}$ , $q_{s}\left(0\right)={\left[\begin{array}{cc} 0.25pi & 0.1pi \end{array}\right]}^{T}$ . The time delay of the forward communication channel and reverse communication channel of the teleoperation system is $T_{m}=T_{s}=0.6s$ .

**Figure 4:**
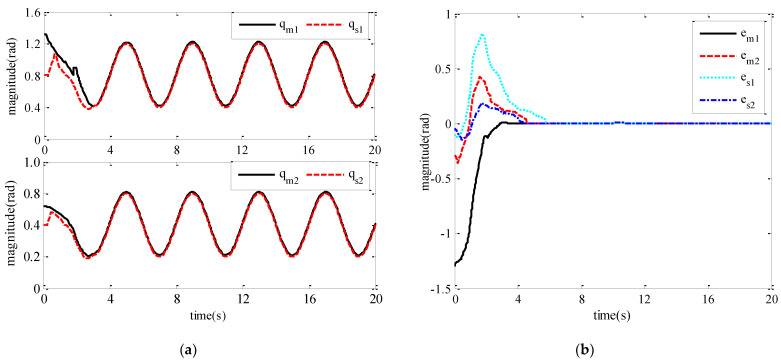
Tracking performance between master and slave robots. (**a**) Tracking of the joint position of the master and slave robots; (**b**) Tracking error of the joint position of the master and slave robots.

**Figure 5:**
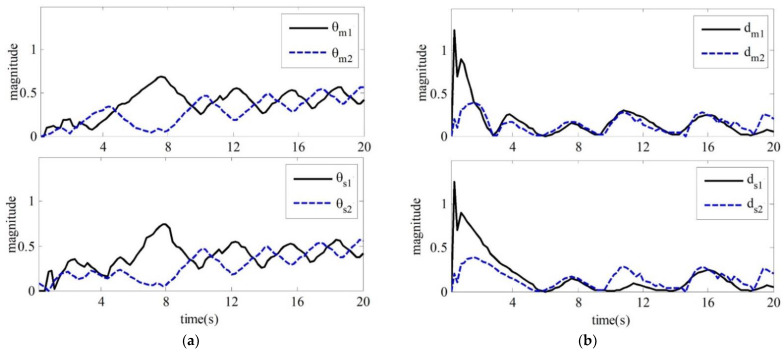
Master–slave robot adaptive parameters ${\overset{\wedge }{\theta }}_{j}$ and ${\overset{\wedge }{d}}_{j}$ . (**a**) Value of ${\overset{\wedge }{\theta }}_{j}$ ; (**b**)Value of ${\overset{\wedge }{d}}_{j}$ .

**Figure 6:**
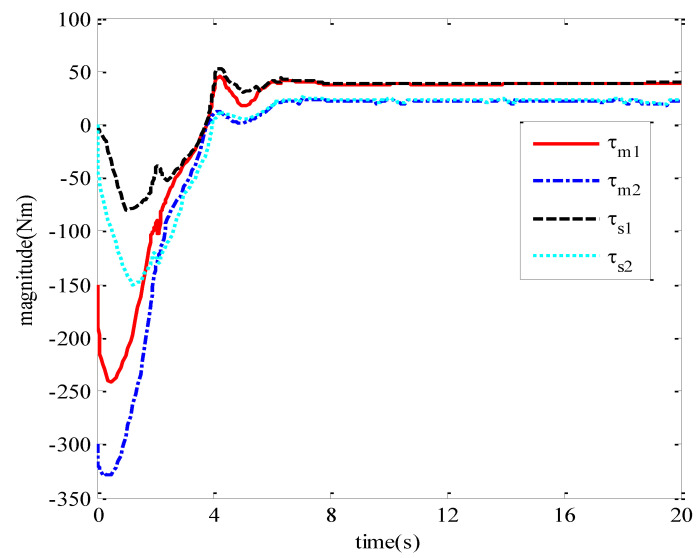
Input torque of master–slave robot joint.

**Figure 7:**
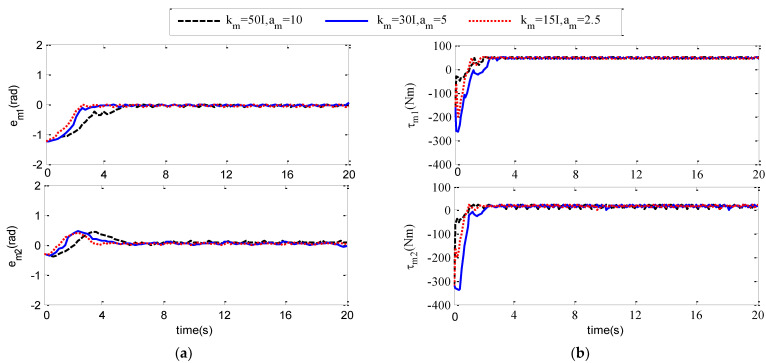
$e_{m}$ and $\tau_{m}$ under different control parameters. (**a**) The joint position tracking error $e_{m}$ of the main robot; (**b**) Joint input torque $\tau_{m}$ of master robot.

**Figure 8:**
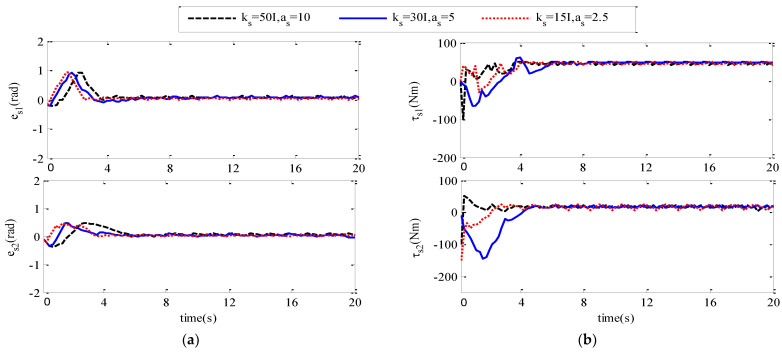
$e_{s}$ and $\tau_{s}$ under different control parameters. (**a**) The joint position tracking error $e_{s}$ of the slave robot; (**b**) Joint input torque $\tau_{s}$ of slave robot.

In the simulation teleoperation system, the controller of the master robot and slave robot adopts Equation (41), which contains adaptive laws of Equations (44) and (45). After repeated debugging, the controller parameters in the teleoperation system are selected as $k_{m}=k_{s}=diag\left(30,30\right)$ , $a_{m}=a_{s}=5$ . Select the parameters $\Lambda_{m}=\Lambda_{s}=5I$ in the sliding mode function. The parameters of the adaptive law are $\gamma_{m}=\gamma_{s}=\lambda_{m}=\lambda_{s}=1$ $\psi_{m}=\psi_{s}=0.2$ , $\upsilon_{m}=\upsilon_{s}=0.01$ . The neural network $W_{m}^{T}\sigma_{m}\left(X_{m}\right)$ , where $X_{m}\in {ℜ}^{10}$ , contains 50 network points, of which the center points are evenly distributed on; the neural network here contains 50, and its center points are evenly distributed on [−10,10] . The simulation results of the experiment are shown in Figure 4, Figure 5, Figure 6, Figure 7 and Figure 8.

The tracking performance between the master robot and the slave robot is shown in Figure 4. Figure 4a shows the track of the joint position of the master robot and the slave robot of the teleoperation system. We can see that the slave robot of the teleoperation system can track the movement of the upper master robot and keep the teleoperation system stable under the condition of the contact motion and the constant time delay of the stable communication channel. Figure 4b shows the error between the joint angle of the master robot and that of the slave robot passing through the reverse channel, and the error between the joint angle of the slave robot and the joint angle signal of the master robot passing through the forward channel.

The values of the adaptive parameters are shown in (a,b) in Figure 5, respectively. Figure 6 shows the input torque diagram of joint 1 and joint 2 of the master robot and slave robot of the teleoperation system.

In order to further study how different controller parameters affect the control performance of the adaptive neural network controller, we carry out simulation experiments, and select different control parameters to compare and analyze the influence of control parameters on the control performance of the controller. The specific experiments are as follows: select three groups of different parameters, respectively, for the following three cases: $k_{m}=k_{s}=50I$ , $a_{m}=a_{s}=10$ ; $k_{m}=k_{s}=30I$ , $a_{m}=a_{s}=5$ ; $k_{m}=k_{s}=15I$ , $a_{m}=a_{s}=2.5$ . The simulation results are shown in Figure 7 and Figure 8.

**Figure 9:**
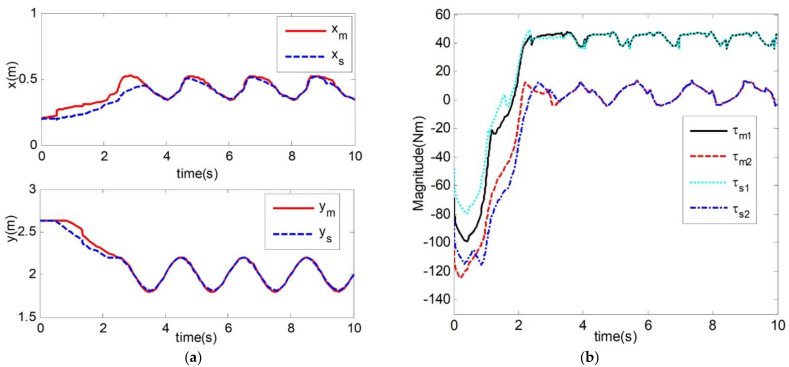
Simulation results. (**a**) Master and slave robot end position trajectory; (**b**) Master and slave robot joint input torque.

Figure 9 shows the results of another method used for the time delay control system, which is an adaptive control of the teleoperation system based on position error structure [24]. Compared with the method mentioned in Figure 9, the teleoperation system studied in this paper also considers the unknown internal friction and external interference of each joint between the master robot and the slave robot. We can observe from it that the position curves of the master robot and the slave robot’s end almost overlap after about 3.5 s in Figure 9a. Adaptive control of the teleoperation system is based on position error structure. Compared with Figure 4a, the position curves of the master robot and the slave robot’s end almost overlap at about 3 s, the method applied in this paper enables the slave robot of the teleoperation system to track the position of the master robot faster, and, with regards to the degree of overlap between the subsequent two curves, the method in this article overlaps better than the method mentioned in Figure 9. The method in this paper can enable the slave robot of the teleoperation system to track the position of the master robot. Figure 9b shows the input torque of joint 1 and joint 2 of the master robot and slave robot of the teleoperation system. Compared with Figure 6, both delay force feedback teleoperation systems can maintain stability.

## 4. Discussion

In this paper, aiming at the dynamic parameter uncertainty, nonlinear parameter uncertainty and time delay of the teleoperation system model linearization, in order to effectively improve the control performance and tracking performance of the teleoperation system, an adaptive neural network controller based on Lyapunov’s method is designed, which is integrated into the position error control structure. The stability of the closed-loop system and the boundedness of the position tracking error convergence are theoretically proved. Finally, the effectiveness of the proposed control scheme is verified by MATLAB Simulink numerical simulation, and the influence of the different controller parameters on the control performance of the system is studied. The effectiveness of our control method is proved by the above simulation data.

The advantages of the proposed controller are as follows [15,16,17]: (1) the RBF neural network is used to approximate the system model with communication delay signal, and the adaptive control method is combined. In this way, the unknown internal friction of each joint of the master–slave robot, the unknown external interference and the constant communication delay in the combined teleoperation system model can be dealt with, and it has good robustness. (2) The control method designed in this chapter contains less online update learning parameters, which reduces the online learning time, thus improving the tracking performance of the system, and is more easily applied to the actual time-delay force feedback teleoperation system. (3) This method not only guarantees the stability of the teleoperation system with time-delay force feedback but also has good control performance.

## 5. Conclusions

From Figure 4, we can see that the slave robot of the teleoperation system can track the movement of the upper master robot, and the teleoperation system can maintain the motion of the upper master robot under the condition of the contact motion and the constant time delay of the communication channel stable [13,14]. Figure 4b shows the error between the joint angle of the master robot and that of the slave robot passing through the reverse channel in the teleoperation system, and the error between the joint angle of the slave robot and the joint angle signal of the master robot passing through the forward channel. From the figure, we can see that the tracking error of the joint position of the master robot and the slave robot can approach 0 in about 5 s. By calculating the average tracking error of the closed-loop constant time-delay force feedback teleoperation system, the average tracking error of the main robot joint 1 is 0.0765 rad, the average tracking error of joint 2 is 0.0521 rad, the average tracking error of slave robot joint 1 is 0.0741 rad, and the average tracking error of joint 2, 0.0612 rad, can be achieved, which shows that the control method in this chapter has good control performance.

The three diagrams from Figure 4, Figure 5 and Figure 6 show that the teleoperation system with time-delay force feedback has good stability and transient performance under the bilateral control method of the teleoperation system designed in this paper.

Figure 7a,b show the position tracking error signal curve and input torque signal curve of the main robot joint 1 and joint 2 of the teleoperation system under three groups of different controller parameters. (a,b) in Figure 4, Figure 5, Figure 6 and Figure 7 show the position tracking error signal curve and input torque signal curve of the teleoperation system from the robot joint 1 and joint 2 under three groups of different controller parameters. From the comparison of the two graphs, it can be concluded that the larger the value of $k_{m},k_{s},a_{m},a_{s}$ , the faster the convergence speed will be, the chattering phenomenon will occur, and the value of the corresponding control torque signal at that moment is greater.

## Figures and Tables

**Table 1:** Symbol meaning.

Meaning	Symbol
The master and slave robots	i(i=m,s)
Joint angular position	$q_{i}\in {ℜ}^{n\times 1}$
Angular velocity	${\dot{q}}_{i}\in {ℜ}^{n\times 1}$
Angular acceleration	${\ddot{q}}_{i}\in {ℜ}^{n\times 1}$
The inertia matrix	$M_{q_{i}}\left(q_{i}\right)\in {ℜ}^{n\times n}$
The Coriolis force and centripetal force matrix	$C_{q_{i}}\left(q_{i},{\dot{q}}_{i}\right)\in {ℜ}^{n\times n}$
Jacobian matrix	$J_{i}\left(q_{i}\right)\in {ℜ}^{n\times n}$
Transposition of the Jacobian matrix	$J_{i}^{T}\left(q_{i}\right)$
The force exerted by the operator on the master robot	$F_{h}\in {ℜ}^{n\times 1}$
The interaction force between the slave robot and the environment module	$F_{e}\in {ℜ}^{n\times 1}$

**Table 2:** Master–slave robot parameters, operator and environment parameters.

$m_{m1}$	$l_{m1}$	$m_{m2}$	$l_{m2}$	$m_{s1}$	$l_{s1}$	$m_{s2}$	$l_{s2}$
0.5 kg	0.6 m	0.5 kg	0.4 m	0.5 kg	0.6 m	0.5 kg	0.4 m
g	$f_{d1}$	$f_{d2}$	$f_{d3}$	$f_{d4}$	$k_{1}$	$k_{2}$	$k_{3}$
$9.81\;m/s^{2}$	1	2	3	3	3	2	4
$k_{4}$	$M_{h}$	$B_{h}$	$K_{h}$	$M_{e}$	$B_{e}$	$K_{e}$	
6	0.2 kg	50 Ns/m	1000 N/m	0.1 kg	20 Ns/m	1000 N/m	

## Data Availability

The data presented in this study are available on request from the corresponding author. The data are not publicly available due to that the experiment at current stage are not at the level to be published.
