# Correction: Wang et al. Adaptive Neural Network Control of Time Delay Teleoperation System Based on Model Approximation. *Sensors* 2021, *21*, 7443

**DOI:** 10.3390/s26061763

**Published:** 2026-03-11

**Authors:** Yaxiang Wang, Jiawei Tian, Yan Liu, Bo Yang, Shan Liu, Lirong Yin, Wenfeng Zheng

**Affiliations:** 1 School of Innovation and Entrepreneurship, Xi’an Fanyi University, Xi’an 710105, China; yaxiang.wang.cn@gmail.com; 2 School of Automation, University of Electronic Science and Technology of China, Chengdu 610054, China; jravis.tian23@gmail.com (J.T.); eevian.liu@gmail.com (Y.L.); yangbo.sd@gmail.com (B.Y.); wenfeng.zheng.cn@gmail.com (W.Z.); 3 Department of Geography and Anthropology, Louisiana State University, Baton Rouge, LA 70803, USA; yin.lyra@gmail.com

## References Correction

In the original publication [1], References 3,5–12,14,16,20,21,25,31,32 were identified as unrelated or inappropriate to the study. These citations have been removed. To provide a more accurate background, three authoritative review articles have been added and are now listed as References 3,5,6 in the revised manuscript.

3. Bhattacharya, S.; Rawat, D. Comparative study of remote surgery techniques. In Proceedings of the 2015 IEEE Global Humanitarian Technology Conference (GHTC), Seattle, WA, USA, 8–11 October 2015; pp. 407–413.
5. Sun, D.; Naghdy, F.; Du, H. Application of wave-variable control to bilateral teleoperation systems: A survey. *Annu. Rev. Control* **2014**, *38*, 12–31.
6. Shahbazi, M.; Atashzar, S.F.; Patel, R.V. A Systematic Review of Multilateral Teleoperation Systems. *IEEE Trans. Haptics* **2018**, *11*, 338–356.

With this correction, the order of some references has been adjusted accordingly.

## Text Correction

The first paragraph of Section 1 has been updated accordingly. The correct paragraph appears below:

Teleoperation robot systems have developed rapidly and been applied to many fields, such as space robots [1], remote surgery robots [2,3], teleoperation mobile robots [4] and so on. The general remote operation robot system mainly includes: a master module, operator module, master controller, communication channel, slave controller, slave, environment and so on. The frame diagram is shown in Figure 1. However, in the actual teleoperation mechanical system, it is difficult to obtain accurate mechanical parameters of the robot, such as mass, length, center of mass or moment of inertia, etc., resulting in the system dynamics parameters (inertia vector matrix, centrifugal force matrix and gravity term matrix) not being accurate, as well as uncertain external interference and mechanical internal friction, which are common in robot workspace control [5]. The complex working environment or the robot’s mechanical structure is, therefore, more complicated or can be destroyed. After modeling using mathematical models, these may be random or time-varying nonlinear functions. Therefore, we cannot accurately establish the mathematical model of the system. That is, the mathematical model of the system contains uncertainty. These problems are often encountered in teleoperation systems, and their manifestations are quite variable. Moreover, the uncertainty of these teleoperation system models not only affects the performance of the system but also makes the entire system unstable [6]. Therefore, how to solve the above problems has been a wide concern in the field of control [7].

There was a typographical error in Section 1.1, Paragraph 1. The “(28)” has been updated to “[28]”. 

The authors state that the scientific conclusions are unaffected. This correction was approved by the Academic Editor. The original publication has also been updated.
